# Complete Genome Sequence of *Streptococcus iniae* 89353, a Virulent Strain Isolated from Diseased Tilapia in Taiwan

**DOI:** 10.1128/genomeA.01524-16

**Published:** 2017-01-26

**Authors:** Hong-Yi Gong, Sheng-Han Wu, Chun-Yao Chen, Chang-Wen Huang, Jenn-Kan Lu, Hsin-Yiu Chou

**Affiliations:** a Department of Aquaculture, National Taiwan Ocean University, Keelung, Taiwan; b Center of Excellence for the Oceans, National Taiwan Ocean University, Keelung, Taiwan; c Department of Life Science, Tzu Chi University, Hualien, Taiwan

## Abstract

*Streptococcus iniae* 89353 is a virulent strain isolated from diseased tilapia in Taiwan. The full-genome sequence of *S. iniae* 89353 is 2,098,647 bp. The revealed genome information will be beneficial for identification and understanding of potential virulence genes of *Streptococcus iniae* and possible immunogens for vaccine development against streptococcosis.

## GENOME ANNOUNCEMENT

*Streptococcus iniae*, a Gram-positive bacterial pathogen originally isolated from Amazon freshwater dolphin (*Inia geoffrensis*) (1), causes disease in both humans (2) and fish (3). In aquaculture, *S. iniae* is a serious marine and freshwater fish pathogen causing mortality and significant economic losses in rainbow trout (*Oncorhynchus mykiss*), tilapia (*Oreochromis* spp.), barramundi (*Lates calcarifer*), red drum (*Sciaenops ocellatus*), flounder (*Paralichthys* spp.), gilt-head seabream (*Sparus aurata*), and red porgy (*Pagrus pagrus*). The virulent strain *S. iniae* 89353 in this study was originally isolated from diseased hybrid tilapia during a disease outbreak in Kaohsiung, Taiwan, in 2000 by Chia-Ben Chao. However, the virulence factors associated with the genome of *S. iniae* 89353 are still unknown.

The genome of *S. iniae* 89353 was sequenced with single-molecule real-time (SMRT) sequence technology by using the PacBio RSII sequencer. The Hierarchical Genome Assembly Process (HGAP) in SMRT analysis was used for *de novo* assembly of total 1,183,191,847 bp from 77,724 sequence reads, with an average length of 15,223 bp (*N*_50_ read length, 20,684 bp). The assembled genome of *S. iniae* 89353 is 2,098,647 bp, with a 1,886,980-bp coding region (89.91%) and a G+C content of 36.8%. The *S. iniae* 89353 genome was predicted to be composed of 2,064 genes, including 1,978 protein-coding sequences (CDSs), 68 tRNAs, and six rRNA operons (16S ribosomal RNA, 23S ribosomal RNA, and 5S rRNA) by NMPDR, SEED-based Rapid Annotations using Subsystems Technology (RAST) version 2.0 server (4). The 1,978 predicted protein-encoding genes belong to 315 subsystems and include 287 genes involved in carbohydrates, 187 genes in protein metabolism, 153 genes in amino acids and derivatives, 116 genes in cell wall and capsule, 102 genes in RNA metabolism, 96 genes in DNA metabolism, 81 genes in cofactors, vitamins, prosthetic groups, and pigments, 70 genes in fatty acid, lipids, and isoprenoids, 64 genes in nucleosides and nucleotides, 54 genes in virulence, disease, and defense, 47 genes in membrane transport, 39 genes in stress response, 36 genes in phosphorus metabolism, 31 genes in cell division and the cell cycle, 25 genes in regulation and cell signaling, 23 genes in iron acquisition and metabolism, 22 genes in miscellaneous, 15 genes in respiration, 10 genes in sulfur metabolism, eight genes in secondary metabolism, five genes in potassium metabolism, two genes in motility and chemotaxis, two genes in the metabolism of aromatic compounds, and one gene in dormancy and sporulation. The complete genome sequence of *S. iniae* 89353 was determined to identify potential virulence genes and candidate immunogens for vaccine development against streptococcosis by comparative genomics analysis with *S. iniae* strains isolated from tilapia (5, 6) and flounder (7, 8).

### Accession number(s).

The complete genome sequence of *S. iniae* 89353 was deposited at GenBank under the GenBank accession no. CP017952.
